# “Asking for help is a strength”—how to promote undergraduate medical students’ teamwork through simulation training and interprofessional faculty

**DOI:** 10.3389/fpsyg.2023.1214091

**Published:** 2023-08-28

**Authors:** Michaela Kolbe, Jörg Goldhahn, Mirdita Useini, Bastian Grande

**Affiliations:** ^1^Simulation Centre, University Hospital Zurich, Zurich, Switzerland; ^2^Department of Management, Technology, and Economics, ETH Zurich, Zurich, Switzerland; ^3^Department of Health Sciences and Technology, ETH Zurich, Zurich, Switzerland; ^4^Institute of Anaesthesiology, University Hospital Zurich, Zurich, Switzerland

**Keywords:** teamwork, healthcare, training, simulation, patient safety, education, TeamSIM

## Abstract

The ability to team up and safely work in any kind of healthcare team is a critical asset and should be taught early on in medical education. Medical students should be given the chance to “walk the talk” of teamwork by training and reflecting in teams. Our goal was to design, implement and evaluate the feasibility of a simulation-based teamwork training (TeamSIM) for undergraduate medical students that puts generic teamwork skills centerstage. We designed TeamSIM to include 12 learning objectives. For this pre-post, mixed-methods feasibility study, third-year medical students, organized in teams of 11–12 students, participated and observed each other in eight simulations of different clinical situation with varying degrees of complexity (e.g., deteriorating patient in ward; trauma; resuscitation). Guided by an interprofessional clinical faculty with simulation-based instructor training, student teams reflected on their shared experience in structured team debriefings. Using published instruments, we measured (a) students’ reactions to TeamSIM and their perceptions of psychological safety via self-report, (b) their ongoing reflections via experience sampling, and (c) their teamwork skills via behavior observation. Ninety four students participated. They reported positive reactions to TeamSIM (M = 5.23, SD = 0.5). Their mean initial reported level of psychological safety was M = 3.8 (SD = 0.4) which rose to M = 4.3 (SD = 0.5) toward the end of the course [T(21) = −2.8, 95% CI −0.78 to-0.12, *p* = 0.011 (two-tailed)]. We obtained *n* = 314 headline reflections from the students and *n* = 95 from the faculty. For the students, the most frequent theme assigned to their headlines involved the concepts taught in the course such as “10 s for 10 min.” For the faculty, the most frequent theme assigned to their headlines were reflections on how their simulation session worked for the students. The faculty rated students’ teamwork skills higher after the last compared to the first debriefing. Undergraduate medical students can learn crucial teamwork skills in simulations supported by an experienced faculty and with a high degree of psychological safety. Both students and faculty appreciate the learning possibilities of simulation. At the same time, this learning can be challenging, intense and overwhelming. It takes a team to teach teamwork.

## 1. Introduction

As of today, teams play an increasingly critical role in healthcare. Healthcare is not only getting more and more specialized, but patients live longer and new technological developments change the way healthcare is provided. The ability to team up and safely work in any kind of healthcare team is becoming a critical asset. Pandemics such as COVID-19 have required healthcare professionals with vastly differing sets of experiences to team up on the spot and learn how to care for newly emerging and changing diseases (Tannenbaum et al., 2020). In contrast with this global development, the education of teamwork skills in healthcare is still in its infancies (World Health Organization, 2021). Teamwork skills are still labeled “soft” and “non-technical” (Hamilton et al., 2019; Kerins et al., 2020; Pollard and Tombs, 2022), although evidence demonstrates that they are everything but “soft” (Nestel et al., 2011; Goldman and Wong, 2020). This dichotomy of clinical vs. non-clinical skills contributes to the minimal emphasis and widely remaining lack of awareness of the importance of teamwork in patient safety in traditional education of healthcare providers (World Health Organization, 2021). Instead, teamwork should be integrated as early as possible in medical education (Banerjee et al., 2016; Chandrashekar and Mohan, 2019).

Training is an effective intervention to improve teamwork skills in healthcare (Hughes et al., 2016; Didwania et al., 2020). Simulation-based training in particular is becoming more and more established in medical education as it allows educators and students to practice and reflect on skills in specialized settings without risking patient safety (Jowsey et al., 2018, 2020). Simulations of clinical teamwork situations provide students with possibilities to reflect on own actions within the context of clinical work. Its particular use for improving interprofessional teamwork skills is growing (Chakraborti et al., 2008; Fox et al., 2018; Hamilton et al., 2019; Sivarajah et al., 2019; Challa et al., 2021; Pollard and Tombs, 2022) and even undergraduate students with limited clinical exposure seem to be able to manage the considerable cognitive load involved in simulation-based learning (Tremblay et al., 2023). However, despite teamwork being part of the learning objectives, it is frequently taught in the context of managing medical emergencies in teams (Weller, 2004; Jowsey et al., 2020; Rouse et al., 2022; Soellner et al., 2022) or in an individual setting (e.g., a single learner performing a tasks with multiple simulated team members and being debriefed individually, Schober et al., 2019). The importance of teamwork skills in healthcare expands beyond emergencies and comes into play in a variety of tasks and team settings such as medical board meetings, handovers or preparing a child for general anesthesia (Foster and Manser, 2012; Schmutz and Manser, 2013; DiazGranados et al., 2014; Taplin et al., 2015; Kolbe and Boos, 2019; Schmutz et al., 2019; Mendoza et al., 2021; Walsh et al., 2021; Zajac et al., 2021; Greilich et al., 2023). Students should be given the chance to “walk the talk” of teamwork by training and reflecting in teams (Arabi and Kennedy, 2022). Our goal was to design, implement and explore the feasibility of a simulation-based teamwork training (TeamSIM) for undergraduate medical students—training in teams—that puts generic teamwork skills centerstage. The goal of this study is to evaluate the feasibility of TeamSIM based on students’ reactions, reflections, and skills.

## 2. Simulation-based teamwork training: TeamSIM

Ten teamwork skills are considered particularly important for working in healthcare teams: (1) recognizing criticality of teamwork, (2) creating a psychologically safe environment, (3) structured communication, (4) closed-loop communication, (5) asking clarification questions, (6) sharing unique information, (7) optimizing team mental models, (8) mutual trust, (9) mutual performance monitoring, and (10) reflection/debriefing (Greilich et al., 2023). We designed TeamSIM to allow medical students to develop, experience and reflect on concepts and strategies for the majority of these teamwork competencies. TeamSIM aims at providing medical students with the possibility to learn principles of working together efficiently, effectively and safely in any interprofessional healthcare teams in a variety of clinical situations, both emergency and routine (Kolb, 1984; Salas et al., 2013; Walsh et al., 2021).

### 2.1. TeamSIM’s pedagogical framework and principles

Based on experiential learning within simulation-based education, TeamSIM is designed for undergraduate medical students. Organized in teams, they are invited to participate and observe each other in simulations of different clinical situation. Guided by an interprofessional clinical faculty with simulation-based instructor training (i.e., nurses, midwifes, physicians, psychologists), student teams reflect on their shared experience in structured team debriefings. They practice essential teamwork skills such as handover communication and speaking up and can experience the translational effects of psychological safety (Pollard and Tombs, 2022; Purdy et al., 2022).

A core pedagogical principle of TeamSIM is single and double-loop learning (Argyris, 2002). Single-loop learning involves learning and refining skills by comparing one’s behavior with practice standards (Argyris, 2002). Here, simulation faculty support learners by teaching and coaching (Fey et al., 2022). Double-loop learning helps learners to identify the frames (i.e., assumptions, beliefs, mental models) that drive their particular behavior (Argyris, 2002). Students may learn that the assumptions they think they hold (i.e., espoused frames, e.g., “teamwork is important”) differ from the assumptions that actually drive their behavior (i.e., actual frames, e.g., actually thinking that “clinical skills are much more important than teamwork skills” and, as a consequence, not engaging in shared pre-briefings to plan their work). Here, simulation faculty supports via facilitation by sharing their observations and points of view and inquiring the students’ point of view (Rudolph et al., 2007, 2008b; Fey et al., 2022).

Single and double-loop learning are represented in SimZones—a system to organize simulation activities based on learners, learning objectives, signal and noise and action, feedback and debriefing. We consider TeamSIM in between SimZone 2 (i.e., acute situational instruction) and 3 (i.e., team and system development, Roussin and Weinstock, 2017). Simulation faculty engages students in coaching and debriefing conversations (Fey et al., 2022). Simulation activities in SimZone 2 and 3 typically involve complex and challenging team tasks and allow learners to deliberately learn from “productive” failure (Sinha and Kapur, 2021). To be able to learn, however, students must feel valued, appreciated and feel that they can share what is on their mind without any repercussions (Edmondson, 1999). This psychological safety is one of TeamSIM’s fundamental pedagogical principles and tracked during TeamSIM’s formative feasibility evaluation (Edmondson, 1999; Rudolph et al., 2014; Johnson et al., 2020; Kolbe et al., 2020; Kostovich et al., 2020; Lackie et al., 2022; Purdy et al., 2022).

### 2.2. Competencies underlying TeamSIM

In Switzerland, the Joint Commission of the Swiss Medical Schools has issued the Principal Relevant Objectives and Framework for Integrated Learning and Education (PROFILES). They explicitly include the ability to work in healthcare teams as learning objective (Michaud et al., 2016). PROFILES displays three interdependent chapters focusing on General Objectives, Entrustable professional activities (EPAs) and the 265 most common clinical situations. TeamSIM covers learning objectives of the General Objectives, which relate to the different roles of physicians as well as several EPAs, which focus on the main tasks a physician must be able to perform autonomously. Specifically, students examine their roles as medical expert, collaborator, scholar, and professional. The EPAs covered here focus on activities that particularly include teamwork.

## 3. Learning environment and format

### 3.1. Learning objectives

**Figure 1:**
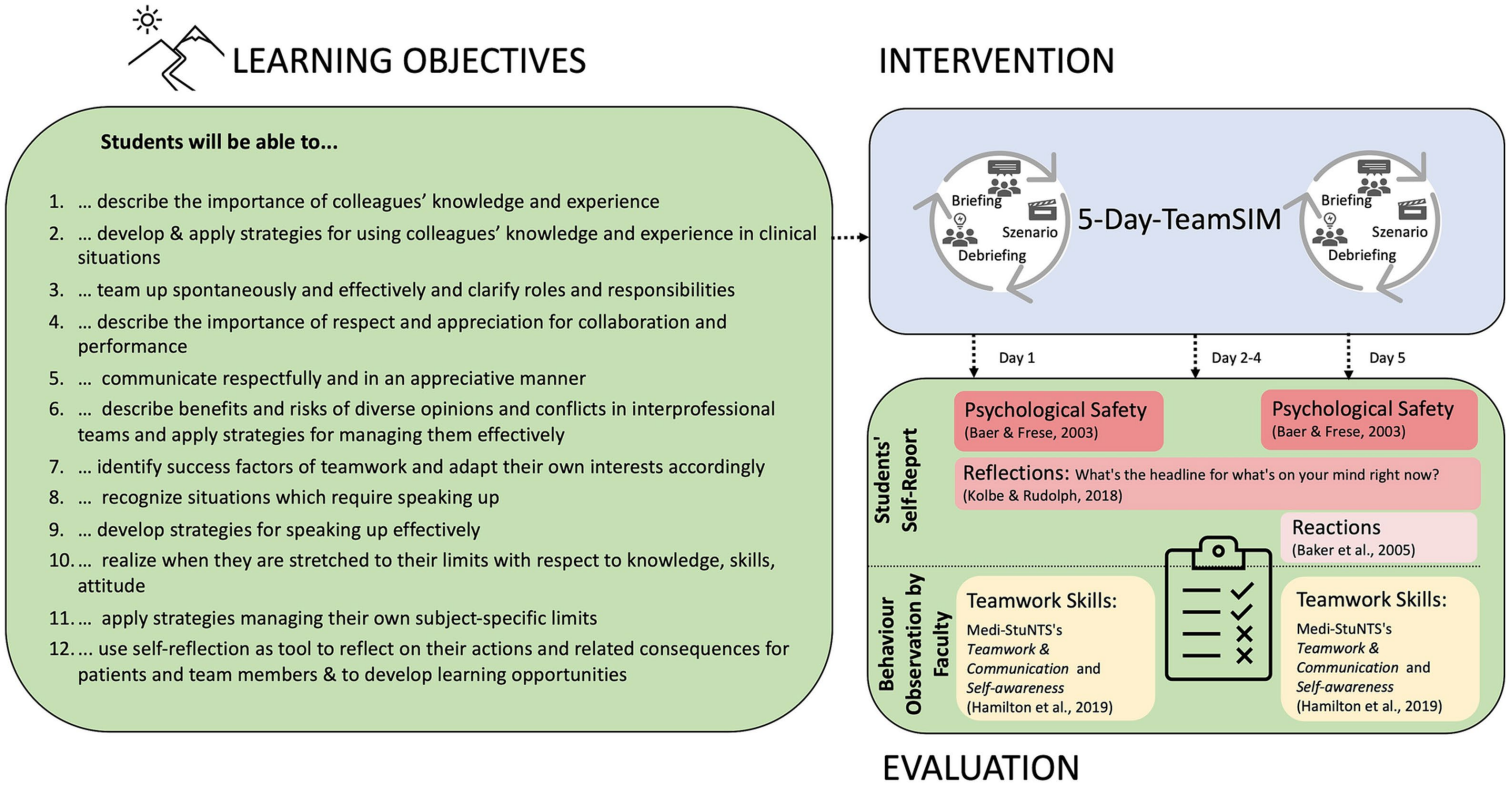
Learning objectives and feasibility evaluation of TeamSIM.

TeamSIM includes 12 learning objectives (Figure 1) around recognition and management of knowledge within teams, teaming up, communicating clearly and respectfully, embracing and managing dissent, voice and listening, asking for help and reflexivity (Larson et al., 1998; Christensen et al., 2000; Baron, 2005; Riskin et al., 2015; Schmutz and Eppich, 2017; Hamilton et al., 2019; Riskin et al., 2019; Schwappach et al., 2019; Long et al., 2020; Jones et al., 2021; Kolbe et al., 2021; Lemke et al., 2021; Rudolph et al., 2021; Bamberger and Bamberger, 2022; DiPierro et al., 2022; Taiyi Yan et al., 2022; Vauk et al., 2022; Greilich et al., 2023; Tannenbaum and Greilich, 2023). Additionally, it provides students with the possibility to reflect on the consequences of teamwork for well-being and performance of healthcare professionals as well as for patient care and safety and on implications for their career management.

### 3.2. Learning environment

Guided by an interprofessional clinical faculty, student teams are invited to reflect on their shared experience in structured team debriefings following each simulated case. Experiencing the transformational effects of team psychological safety and practicing teamwork skills in this learning environment is the core of TeamSIM (Roussin et al., 2014; Rudolph et al., 2014; Edmondson and Bransby, 2023). Simulation is a powerful teaching tool and psychological safety and high-quality facilitation are important. We deliberately aimed at establishing a faculty team of experienced simulation educators (rather than student teachers) who are able to guide students respectfully through challenging simulation exercises, debriefings and deal with difficult situations (Grant et al., 2018; Jowsey et al., 2020; Kolbe et al., 2020). They work as physicians, nurses, midwifes, psychologists, and an airline pilot, and typically train their peers rather than students. For TeamSIM, they underwent specific faculty development: the course directors provided detailed orientation on TeamSIM’s learning objectives and curriculum; coordinated objectives and simulation across sessions, scheduled faculty briefing and debriefings, reviewed each of the eight different simulation sessions’s modules, and conveyed their commitment to psychological safety. If possible, faculty conducts debriefs each day over lunch during TeamSIM.

### 3.3. Pedagogical format

TeamSIM is designed as week-long course and is open to all third-year medical students of a new bachelor on human medicine at ETH Zurich, Switzerland (Weissmann et al., 2020). We invite students to “walk the talk” of teamwork by training teamwork in teams.

**Figure 2:**
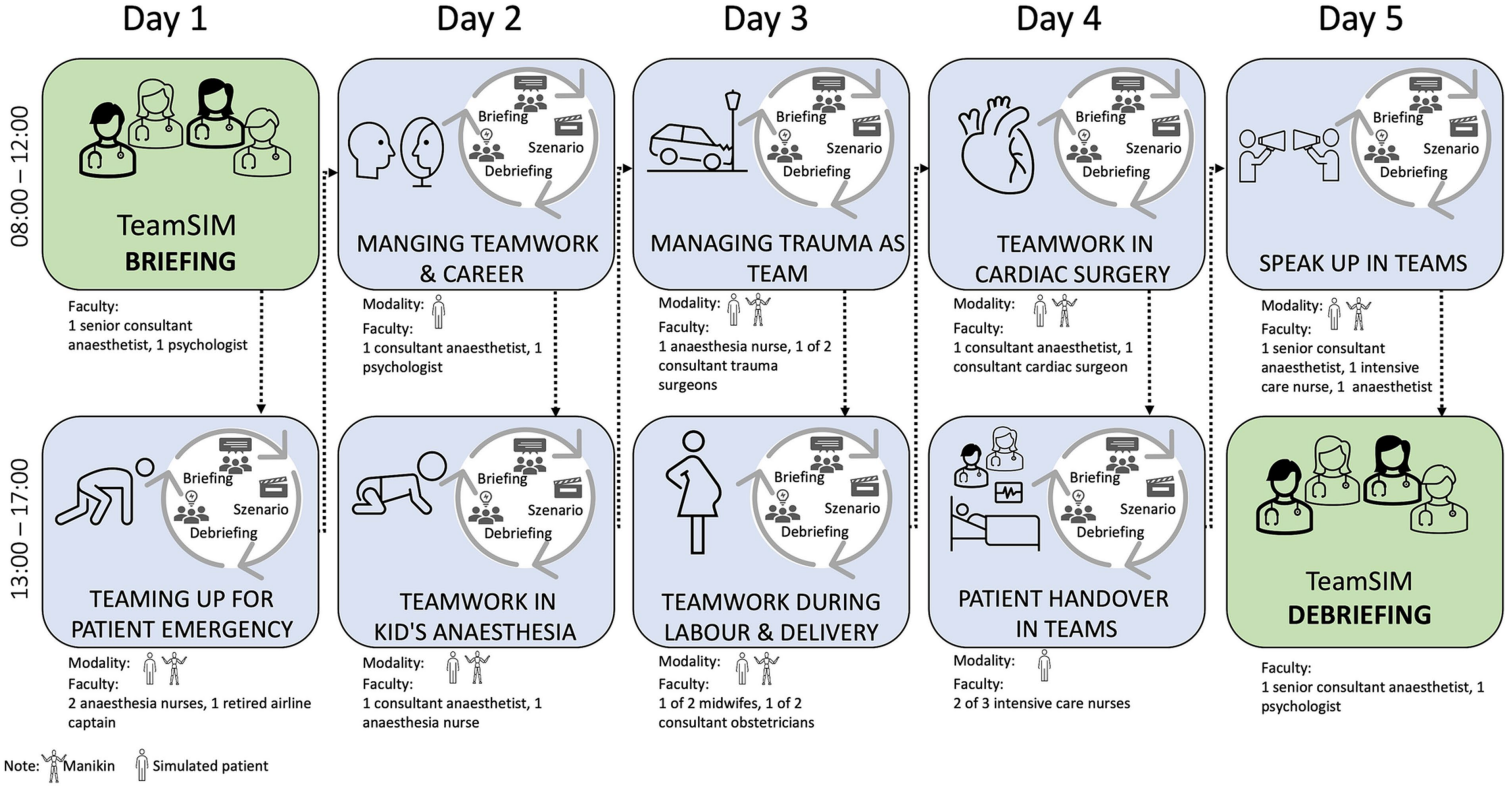
TeamSIM sample curriculum for team 1 with simulation sessions, modality, and faculty.

We organize students in teams of 11 to 12 people. They remain in their respective team for the full week and participate in eight, in-person, four-hour-simulation sessions representing different clinical situations with varying degrees of complexity (e.g., deteriorating patient in ward; trauma; labor and delivery, Figure 2). Due to logistical reasons, each of the teams follows a slightly different schedule: Team 1 starts with “Teaming up for patient emergency” and ends with “Speak up in teams” (Figure 1) while Team 2 starts with “Managing teamwork and career” and ends with “Teaming up for patient emergency,” etc. Each of the eight 4-hour simulation sessions includes participation in two to three rounds of briefing, simulated case, and debriefing (Figure 2). Students can practice teamwork skills such as leadership from three different perspectives. For example, they can lead the team (1st-person practice), be led by a team member (2nd-person practice) and observe team leadership and followership from the outside (3rd-person practice, Chandler and Torbert, 2003). That is, depending on the case, four to six of the students actively participate while their peers observe them; roles are switched in the subsequent case. The simulated cases are developed by the faculty teaching the respective session (Figure 2).

For the debriefings, the faculty follows the Debriefing with Good Judgment (Rudolph et al., 2007, 2008a) and TeamGAINS (Kolbe et al., 2013) approaches. They use their observations of the students’ actions during the scenarios to provide feedback, inquire their perspectives and discuss different approaches with all team members. During both briefing and debriefing faculty focuses on selected learning objectives. For example, during the session “managing trauma as team,” the faculty introduces and discusses tools for developing the learning objectives #2 (develop & apply strategies for using colleagues’ knowledge and experience in clinical situations), #3 (team up spontaneously and effectively and clarify roles and responsibilities), #10 (realize when they are stretched to their limits with respect to knowledge, skills, attitude), and #11 (apply strategies managing their own subject-specific limits). In addition, the faculty adapts their focus depending on the students’ needs (Cheng et al., 2016). For example, students can re-do or practice certain team actions.

We introduce all students to the course during the formal TeamSIM briefing on Monday morning. Using Zoom (Zoom Video Communications, Inc., San Jose, United States), we discuss expectations, course of events, learning objectives, confidentiality, roles, and logistic details to provide orientation and contribute to a psychologically safe learning environment (Rudolph et al., 2014; Kolbe et al., 2020). We introduce simulation as a teaching tool, reflect on its advantages and limitations, provide recommendations for how to engage in simulation and demonstrate our commitment to respecting students and their perspective. We then invite students into breakout groups in their respective teams to brief themselves and ask them to develop a set of guiding principles for their team (Mathieu and Rapp, 2009). Simulation sessions start Monday afternoon and end Friday morning. TeamSIM ends with a formal TeamSIM debriefing on Friday afternoon when we invite all teams back into Zoom to review TeamSIM. In particular, we ask all student teams to formally debrief themselves based on a structured tool adapted to TeamSIM (Welch-Horan et al., 2021).

Students’ actions are not graded. To pass the course, 90% attendance of the sessions is required.

## 4. Feasibility evaluation of TeamSIM and its evaluation model

Our goal was to explore the feasibility of conducting TeamSIM and its evaluation model. As the course is quite intense with a considerable number of students, faculty, learning objectives, and simulation operations, we intended to investigate the practicability of a pre-post evaluation of each student by the faculty who already has a high workload. The data we collected as described below was merely used for this purpose, treated as confidential, and not reported back to the students. (Students receive immediate feedback as part of the debriefings during each simulation session.)

### 4.1. Methods

#### 4.1.1. Study design and ethics

Our intention was to explore the feasibility of evaluating TeamSIM with the a pre-post, mixed-methods design which required both students and faculty to collect data (Figure 1). We conducted TeamSIM and collected feasibility evaluation data from 13 March until 17 March 2023 at the Simulation Centre of the University Hospital Zurich, Switzerland. The ethics committee of the canton of Zurich, Switzerland granted this study exemption (Registry no. 2023-00194). Study participation was voluntary and participants’ consent was obtained at the time of enrolment.

#### 4.1.2. Sample

Ninety four third-year medical students participated in TeamSIM; 53 students (56.4%) were female, 41 (43.6%) male. We randomly assigned students to eight teams of 11 to 12 students and provided each team with a rotation time table. A pool of 23 experts participated as faculty training each of the teams participating in the simulation sessions with 2 to 3 faculty members. Nine faculty members (39.1%) had a background in anesthesiology, 4 (17.4%) in intensive care, 2 (8.7%) in traumatology, 4 (17.4%) in labor and delivery, 1 (4.3%) in cardiac surgery, 2 (8.7%) in psychology, and 1 (4.3%) in commercial aviation.

#### 4.1.3. Measures

##### 4.1.3.1. Students’ reactions to TeamSIM

At the end of the final simulation session we measured students’ reactions to TeamSIM using a German version of a scale measuring trainee’s reactions to the training (Baker et al., 2005; Kolbe et al., 2013). This scale contained nine items which students rated on a 6-point Likert scale ranging from 1 (strongly disagree) to 6 (strongly agree). Sample items were “The training was an effective use of my time” and “The training was well organised.” In addition, we asked students to respond to four open-ended questions: “What did you particularly like?,” What did you not like?,” “What was your most important learning experience?,” “What do you need to apply the skills learned in this course?”

##### 4.1.3.2. Perceptions of psychological safety

After the introduction to TeamSIM as well as after final debriefing session, we measured psychological safety by administering six items from the validated German translation (Baer and Frese, 2003) of Edmondson’s (1999) team psychological safety scale: (1) “Everyone will be (was) able to bring up problems and tough issues”; (2) “No one would (did) deliberately act in a way that undermines my efforts”; (3) When someone makes (made) a mistake it will be (was) always held against him/her”; (4) “Some people will be (were) rejected for being different”; (5) “Others will (did) value and utilize my unique skills and talents”; (6) “It will be (was) difficult to ask others for help.” Items number 3, 4, and 6 were reverse coded to mitigate response set bias. Items were rated on a 5-point Likert scale ranging from 1 (strongly disagree) to 5 (strongly agree).

##### 4.1.3.3. Reflections

Via experience sampling we tracked what captivated, concerned or transformed students and faculty as they moved through TeamSIM (Larson and Csikszentmihalyi, 1983; Kerins et al., 2020). We applied a modified “Headline”-method (Kolbe and Rudolph, 2018): after each simulation session, students were invited to access an online, two-minute free writing task via QR-code. The writing task was entitled “headline” and included the following open-ended question: “What is the headline for what is on your mind right now?” and the prompt “Headline:,” followed by a blank line indicating participants should answer the question with a few words only.

##### 4.1.3.4. Teamwork skills

TeamSIM faculty aimed to assess teamwork skills using two skill categories of Medi-StuNTS (Hamilton et al., 2019). Medi-StuNTS is a behavioral marker system designed to assess “non-technical” skills of medical students. It comprises of five skill categories: *situation awareness*, *decision-making and prioritization*, *teamwork and communication*, *self-awareness* and *escalating care*. For the purpose of evaluating TeamSIM, we selected *teamwork and communication* and *self-awareness* as relevant skill categories because they appropriately represented TeamSIM’s learning objectives (Table 1). For each skill category, Medi-StuNTS provides three skill elements and respective positive and negative behavioral markers (Hamilton et al., 2019). The skill category teamwork and communication includes the elements (1) establishing a mental model, (2) demonstrating active followership, and (3) patient involvement. The skill category self-awareness includes the elements (1) role awareness, (2) coping with stress, and (3) speaking up (Table 1). Faculty were asked to rate students on a 5-point Likert scale ranging from 1 (poor performance, threatens patient safety, improvement required) to 5 (excellent performance, a positive example for others, Hamilton et al., 2019). A study testing Medi-StuNTS validity and reliability found evidence for discriminatory validity (e.g., experts scoring better than intermediates who scored better than novices) and inter-rater reliability (e.g., disagreement of more than one point in less than one-fifth of cases, Phillips et al., 2021). Medi-StuNTS was designed to be used with minimal training (Hamilton et al., 2019). MK discussed Medi-StuNTS’ content and use with the TeamSIM faculty 1 week prior to TeamSIM start.

**Table 1:** Teamwork skills rated by TeamSIM faculty using Medi-StuNTS categories teamwork and communication and self-awareness after the first (pre-test) and last (post-test) simulation session.

	Pre-test *N* = 52 (55.3%)			Post-test *N* = 61 (64.9%)		
	M	SD	Not observable (*n*)	M	SD	Not observable (*n*)
**Category teamwork and communication**						
Establishing a mental model	3.84	0.83	3	4.15	0.41	2
Demonstrating active followership	3.75	0.84	4	4.17	0.46	2
Patient involvement	3.57	0.85	17	4.18	0.44	12
Role awareness	3.91	0.75	5	4.21	0.49	5
Coping with stress	3.56	0.81	16	4.13	0.46	16
Speaking up	3.70	0.87	6	4.09	0.35	7

#### 4.1.4. Data collection

We created online versions and respective QR access codes of all measures and placed them either on the walls of the training rooms or provided the instructors with them. Both students and instructors could access the measures with their smart phones. Upon the start of the course, we verbally provided all students with information on course evaluation, uploaded detailed information and the consent form on their online learning platform as well as handed them out prior to the start of their first simulation session. We instructed the faculty to support students accessing the evaluation measure. We also asked them to assess teamwork skills at the beginning and end of the course and discuss the Medi-StuNTS with them.

#### 4.1.5. Data analysis

We conducted statistical analysis for trainee reactions, psychological safety, and teamwork skills with SPSS V.26 software (IBM, Armonk, NY, United States). The statistical tests were two-sided using 0.05 as the threshold for statistical significance.

We analyzed responses to the open-ended training reaction questions via applying a multistep, thematic analysis to identify evident topics (Miles and Huberman, 1994; Braun and Clarke, 2006). We considered each response one analytic unit. Following procedures for linking inductive and theory-driven coding we started inductively for each of the four open-ended questions by reviewing response after response and generating a list of rough categories in an open-coding process (Boyatzis, 1998; Fereday and Muir-Cochrane, 2006). We subsequently reviewed rough categories and identified clusters of categories which we used to analyze all responses. We determined absolute frequencies for the resulting categories.

For the headline reflections, we coded the original headlines based on an analytical approach reported for similar data (Kolbe and Rudolph, 2018): we assigned each headline to one or more of five themes: (1) metacognitions of one’s learning process (i.e., statements on monitoring one’s learning progress), (2) evaluations of sessions and performances (i.e., critically reviewing a particular session or how something worked), (3) notes to self (i.e., reflections on specific concepts introduced during TeamSIM), (4) anticipations of applying the learnt skills in the future (i.e., predicting how particular competencies would be used in the future), and (5) emotions in the learning process (i.e., affective statements). We determined absolute frequencies for the resulting groups of codes. We illustrated selected headline reflections using Graphpad.^1^

### 4.2. Results

Of the 94 students participating in TeamSIM, 81 (86.2%) responded to the pre-psychological safety measurement; 45 (47.9%) students completed the post-psychological safety and training reactions survey, 22 (23.4%) of which we could match.

#### 4.2.1. Reactions to TeamSIM

**Figure 3:**
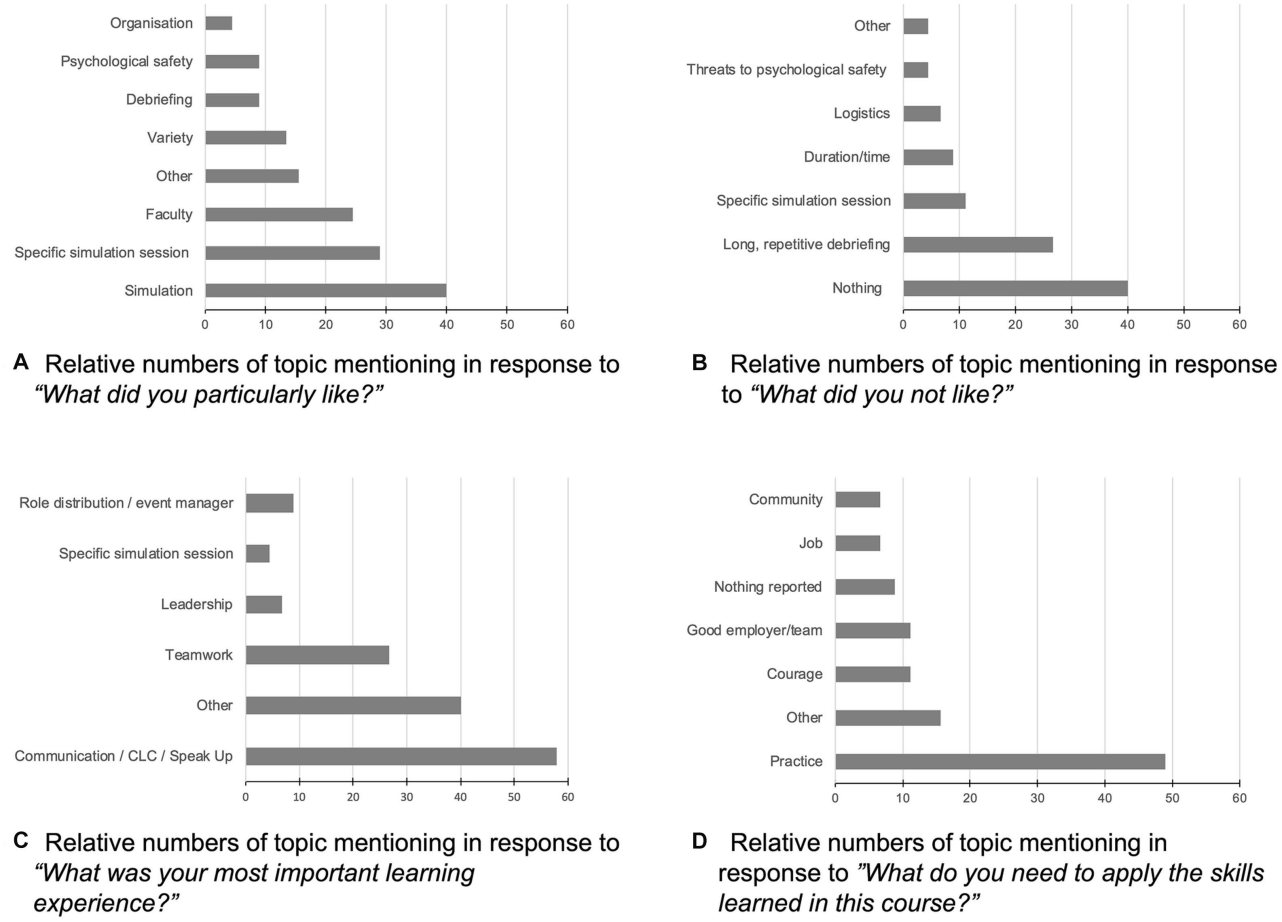
**(A–D)** Relative numbers of topic mentioning in response to four open-ended training reaction questions (*n* = 45). CLC, closed loop communication.

Students reported positive reactions (α = 0.87) to TeamSIM (M = 5.23, SD = 0.5). In response to what the students particularly liked, the three most frequently mentioned topics were the *simulation method* as such, specific *simulation sessions*, and the *way the faculty engaged with them* (Figure 3A). In response to what they did not like about TeamSIM, the three most frequently mentioned topics were *nothing*, *long, repetitive debriefings*, and specific *simulation sessions* (Figure 3B). As their most important learning experience, students reported in particular communication such as *closed-loop communication* and *speaking up*, *teamwork and leadership*, *role distribution*, and a variety of other insights such as “not yet knowing is okay if one knows how to get help,” “thinking out loud,” “admitting one’s fallibility,” or “asking for help is a strength” (Figure 3C). In response to what they might need to apply the skills learned in TeamSIM, students mentioned *practice*, *courage*, a *“good” employer or team* and a variety of other factors such as community and team orientation (Figure 3D).

#### 4.2.2. Perceptions of psychological safety

On a scale from 1 to 5, students’ mean initial reported level of psychological safety (α = 0.44) was M = 3.8 (SD = 0.4). At the end of the course, this level (α = 0.53) rose to M = 4.3 (SD = 0.5). For the *n* = 22 students for whom we could match pre and post responses we found a significant increase in perceived psychological safety [T(21) = −2.8, 95% CI −0.78 to −0.12, *p* = 0.011 (two-tailed)].

#### 4.2.3. Reflections

**Figure 4:**
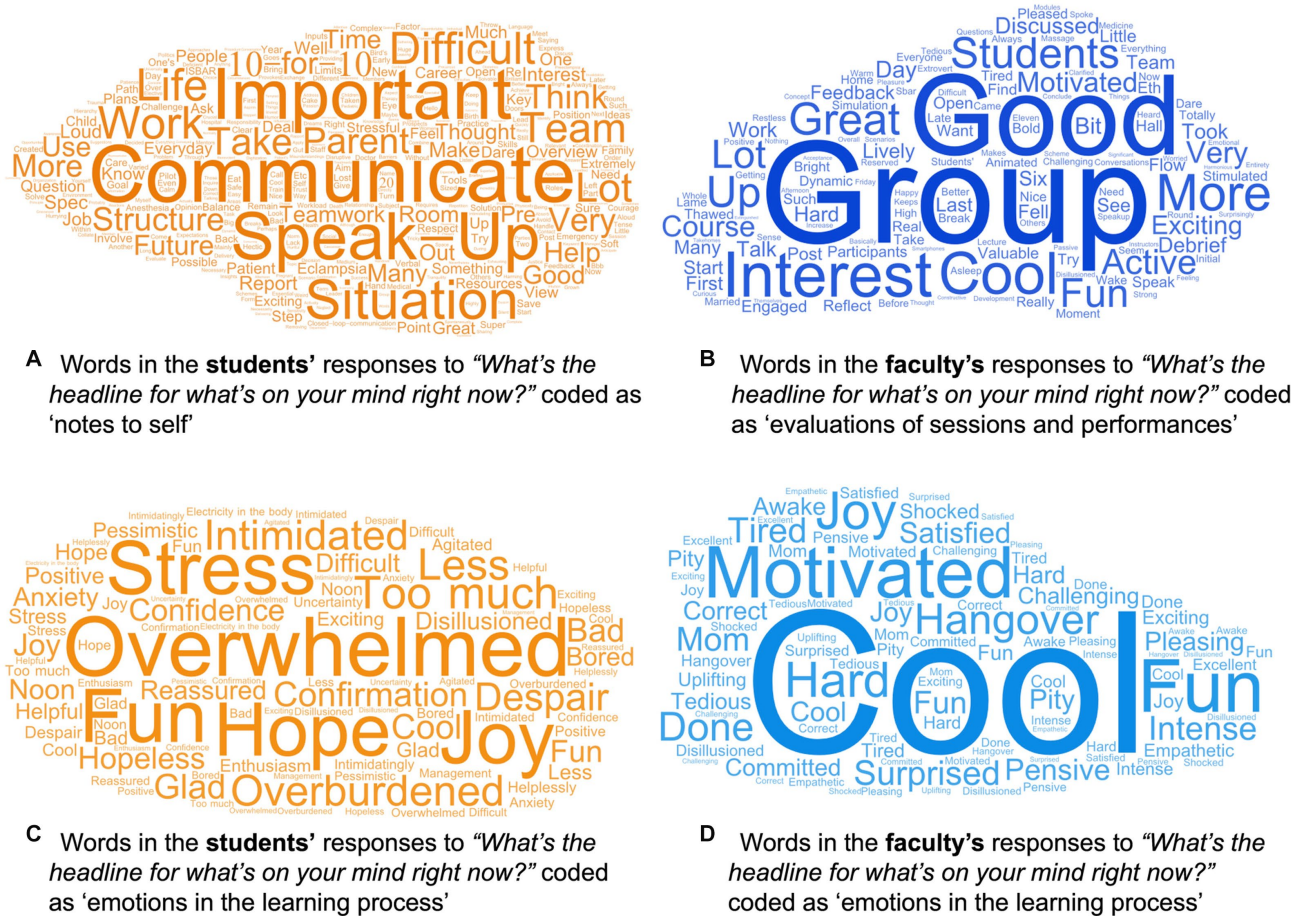
Quantitative graphic representations of the words in the students’ headline reflections coded as “notes to self”(*n* = 181) **(A)** and “emotions in the learning process” (*n* = 32) **(C)** and in the faculty’s headline reflections coded as “evaluations of sessions and performance” **(B)** and “emotions in the learning process” (*n* = 34) **(D)**. More frequently used expressions are represented by larger front sized. Common filler word (i.e., the, is, and) were excluded. The graphics were created using WordArt.com (accessed on 05 April 2023).

We obtained *n* = 314 headline reflections from the students and *n* = 95 headline reflections from the faculty. For the students, the most frequent theme assigned to their headlines was *notes to self* (57.6%) which involved students’ reflections on the concepts taught in the course such as closed-loop communication, speaking up and “10 s for 10 min” (Figure 4A). Other themes of student headlines were *evaluations* (33.4%), *emotions* (10.2%), *anticipations* (4.8%), and *metacognitions* (3.5%). For the faculty, the most frequent theme assigned to their headlines was *evaluation* (61.1%), i.e., reflections on how their simulation session worked for the students or how students seemed to react to the simulation (Figure 4B). Other themes of faculty headlines were *emotions* (35.8%), *notes to self* (15.8%), *metacognitions* (9.5%), and *anticipations* (1.1%). Looking at emotions, for students these emotions were mixed, ranging from joyful to overwhelmed (Figure 4C). For the faculty, the reflected emotions were rather positive, in particular seeing the students improve over the course of TeamSIM (Figure 4D).

#### 4.2.4. Development of teamwork skills

For the rating of teamwork skills, the faculty was able to rate the selected teamwork skills for 52 (55.3%) students immediately following the first TeamSIM simulation session and for 61 (64.9%) students immediately following the final TeamSIM simulation session (Table 1). On a scale from 1 to 5, faculty rated the students initial teamwork skills from M = 3.56 (SD = 0.81) to M = 3.91 (SD = 0.75). At the end of the course, these values rose to M = 4.09 (SD = 0.35) to M = 4.21 (SD = 0.49). Due to challenges in matching students’ pre and post values we refrained from performing inferential statistical analysis.

## 5. Discussion on the practical implications, objectives, and lessons learned

Our goal was to design, implement and evaluate the feasibility of a simulation-based teamwork training—TeamSIM—for medical students. Based on experiential learning within simulation-based education, TeamSIM aims at providing students with the possibility to learn principles of working together efficiently, effectively, and safely in interprofessional healthcare teams. In what follows, we discuss the effectiveness of TeamSIM, challenges, constraints, limitations, and highlight our lessons learned.

### 5.1. Effectiveness of TeamSIM

The feasibility evaluation data suggests that students reacted rather positively to participating in activities simulating their future work with each other. They seemed to engage in in-depth examination of their approaches to teamwork. Students’ teamwork skills seemed to improve and their take-aways indicate specific teamwork capabilities. At the end of the course they felt more psychologically safe than at the start. This is an important finding because simulation is an intense teaching tool which involves social risk-taking and, thus, high level of psychological safety (Edmondson and Bransby, 2023). Importantly, the psychological safety that emerges during the simulation may leak into other fields and transfer to clinical practice (Pollard and Tombs, 2022; Purdy et al., 2022). Although our cross-sectional data prevents us from drawing an empirical conclusion, we assume that the reported learnings, improved teamwork skills, and positive reactions are related to the psychologically safe learning culture. While the students’ seemed to have benefited from a learning environment with a high degree of psychological safety, TeamSIM’s week-long intensity seemed to have asked a lot of their perseverance. The emotions reported by students (Figure 4C) suggest that TeamSIM was—beside being “fun” and “joyful”—“overwhelming” and “too much” at points. While this is normal in most complex, simulation-based training (Kolbe and Rudolph, 2018; Keskitalo and Ruokamo, 2021), distributing TeamSIM over a few weeks rather than 1 week might have provided students with more possibilities to digest their learning. However, given the logistical constraints of the medical curriculum and simulation operations, re-building the TeamSIM infrastructure once a week over a few weeks seems daunting.

### 5.2. Interprofessional faculty and their development

According to the evaluation data, the students appreciated the teaching, coaching, and facilitation by the interdisciplinary and interprofessional simulation faculty whom they perceived as very engaged and committed. This is to some degree reflected in TeamSIM faculty’s reflections which indicated their ongoing concern about the effectiveness of their educational interventions. In our experience, working with this faculty prior to the course was critical and a necessary ingredient: multiple disciplines and professions went along with multiple approaches to simulation; establishing and maintaining a shared mental model of TeamSIM was important and required various faculty development measures (Cheng et al., 2020; Kolbe et al., 2020; Kostovich et al., 2020; Roze des Ordons et al., 2022).

### 5.3. Challenges and constraints

We experienced three particularly challenges. First, designing, planning, coordinating, and conducting TeamSIM involved effort with respect to course curriculum design and coordination of faculty. In addition to preparing the simulation space, equipment and designing sessions, we needed to recruit and develop the interdisciplinary, interprofessional, clinical faculty. Their availability and willingness to make time and engage in this course in their busy clinical schedules was crucial for its success (Fox et al., 2018).

Second, the complexity of TeamSIM and our deliberate choice to engage an experienced, clinical faculty rather than student peer coaches made this course expensive. Simulation-based education is considered a privilege (Lillekroken, 2020; Mosher et al., 2021). While we think that high-quality education will create long-lasting value, we are aware that finding ways to establish TeamSIM’s sustainability will be challenging. We all did, however, consider the significant investment of time and financial resources also as in investment in the faculty’s educational careers. According the headline reflections, the faculty enjoyed teaching this course.

The third challenge involved a potential mismatch of expectations and experience: while the faculty was highly trained in working with clinicians and aware of the importance of reflecting on practice, students seemed to struggle at points with the expected “amount” of reflection and the difficulties of the cases. This might be a common struggle in simulation-based education (Loo et al., 2018), particularly for students (Jowsey et al., 2020). Meeting their various needs for instruction vs. reflection was challenging, and likely reflects variances in their own personal development (Kerins et al., 2020). More in-depth research on how to support students while they learn to embrace reflecting on their actions will be helpful.

### 5.4. Limitations

Our feasibility evaluation of TeamSIM revealed limitations. First, we were not able to collect as much evaluation data as planned. We experienced that performing evaluations (i.e., inviting students again and again to complete surveys and headline reflections, rating teamwork behavior of multiple students, each team following a slightly different schedule) added another layer of workload for the faculty and resulted in a lack of interrater reliability data, low response rates, and dropouts which limit the generalizability of our results. We have learned we should more deliberately plan for collecting complex yet important evaluation data (e.g., engaging additional raters, collecting videos and performing the rating based on videos, peer-observation with pre-trained peers, additional evaluation training, etc.). Second, our emphasis on anonymity limited our ability to track individual students’ over time; matching pre and post measures was challenging and in many cases not possible. It also prevented us from conducting multilevel analysis which would have been required because students were nested in teams (Raudenbush and Bryk, 2002). In addition, it prevented us from exploring effect differences between simulation sessions. Third, we did not perform reliability checks for the qualitative data analysis and the α-values of the psychological safety scale were rather low, again limiting the validity of our evaluation findings. Finally, in designing, conducting and evaluating TeamSIM we did not yet factor in potential cultural differences in both students and faculty, nor did we reflect on aspects of equity, gender and inclusion, which are significant limitations and call for change in future TeamSIM iterations (Palaganas et al., 2021; Purdy et al., 2023).

### 5.5. Lessons learned

The lessons learned from designing, conducting and evaluating the feasibility of TeamSIM are threefold: first, it takes a team to teach teamwork: a team of interprofessional faculty that embraces simulation-based learning and the psychological safety it requires. Second, both students and faculty appreciated the learning possibilities of simulation-based education, in particular for learning teamwork skills. In line with other research, this project endorses simulation as a teaching method that enables students to experience the complexity of interprofessional teamwork in healthcare and to try out and reflect on different approaches for managing this complexity that work for them. As one of the students remarked, it had helped them to develop “cornerstones in midst of the chaos.” At the same time, this learning can be challenging, intense and overwhelming. Importantly, it should be considered in the context of how psychologically safe the students felt during training. Simulation-based training is a powerful tool; without psychological safety it may significantly impede students’ capacity to learn and develop professional identities (Rudolph et al., 2013; Purdy et al., 2022; Edmondson and Bransby, 2023). Thus, while we think that versions of TeamSIM might be useful for training students of other healthcare professions, we strongly recommend to put high emphasis on establishing and maintaining psychological safety. For example, providing orientation about expectations and learning objectives, engaging learners in a sort of “fiction contract,” caring about logistic details, conveying respect for learners and concern for their psychological safety, and maintaining awareness of the dynamics of psychological safety are helpful actions (Rudolph et al., 2014; Kolbe et al., 2020; Somerville et al., 2023). Third, while teamwork in healthcare may involve a somewhat stable set of skills (Greilich et al., 2023), the way students learn may constantly change with their exposure to an increasingly digital world (Balmaks et al., 2021). The pedagogical format of TeamSIM may need to adapt as well. Finally, evaluating such a complex and intense simulation-based teamwork requires additional preparation. In our view, inspite of the involved effort, simulation-based teamwork trainings such as TeamSIM are a valuable contribution to the teamwork capabilities of our future healthcare workforce.

## Data availability statement

The original contributions presented in the study are included in the article/supplementary material, further inquiries can be directed to the corresponding author.

## Ethics statement

The study was ruled exempt by the Kantonale Ethikkommission Zürich. The studies were conducted in accordance with the local legislation and institutional requirements. The participants provided their written informed consent to participate in this study.

## Author contributions

MK and BG designed TeamSIM. MU and JG oversaw its integration into the bachelor of human medicine’s overall curriculum. MK designed the evaluation and performed the data analysis and drafted the manuscript. MK and BG lead the data collection. All authors contributed to the article and approved the submitted version.

## Funding

Open access funding was provided by ETH Zurich.

## Conflict of interest

MK, JG, MU, and BG are faculty of ETH Zurich where the curriculum evaluated in this study is offered. JG is the director of medical studies at ETH Zurich. MU leads the curriculum development of medical studies at ETH Zurich. MK and BG are directors of the TeamSIM course and faculty of the Simulation Centre of the University Hospital Zurich.

## Publisher’s note

All claims expressed in this article are solely those of the authors and do not necessarily represent those of their affiliated organizations, or those of the publisher, the editors and the reviewers. Any product that may be evaluated in this article, or claim that may be made by its manufacturer, is not guaranteed or endorsed by the publisher.

## Footnotes

^1^www.graphpad.com
